# Supplementary material for: Sugar-Pucker Force-Induced Transition in Single-Stranded DNA
Source: Int J Mol Sci. 2021 Apr 29;22(9):4745. doi: 10.3390/ijms22094745 (PMC8124619; doi:10.3390/ijms22094745)
Supplement: Supplementary file 1 [file ijms-22-04745-s001.zip › ijms-1193567-supplementary.pdf]

Supplementary material: Sugar-pucker force-induced transition in single-stranded DNA

X. Viader-Godoy[1,2], M. Manosas[1], and F. Ritort[1]

[1]Departament de Física de la Matèria Condensada, Universitat de Barcelona, Diagonal 647, 08028 Barcelona, Spain
[2]Department of Physics and Astronomy, University of Padova, via Marzolo 8, 35131, Padova, Italy

April 20, 2021

1 DNA hairpin synthesis

The short hairpin (SH) was synthesized following the protocol in Ref. [1] (named as CD4L20). The oligonucleotide forming the 3' end of the hairpin was labelled with a digoxigenin tailing. After a purification step (*QIA Nucleotide removal kit*), all the oligonucleotides forming the hairpin (Table 1) were annealed by starting at a high temperature (70 °C) and 1 °C was decreased every minute until room temperature was reached. The hairpin was next ligated using the T4 DNA ligase (New England Biolabs) in an overnight reaction (16 °C).

The long hairpins (LHs) were synthesized following a procedure based either on a PCR amplification of a dsDNA segment or digesting a segment of the linearized λ-DNA. H700, H964, H4452 and H13680 were prepared as described in Refs. [2] and [3]. Finally, H1904 and H7138 were synthesized following the protocol in Ref. [3], changing the restriction enzyme for the digestion step: *EcoRI* (New England Biolabs) for H7138, and *BspHI* (New England Biolabs) for H1904. The sequences of the oligonucleotides used for preparing the DNA hairpins are given in Sec. 2. Fig. 1(a)-(b) shows an scheme of the molecular construct for the SH and LH hairpins, respectively.

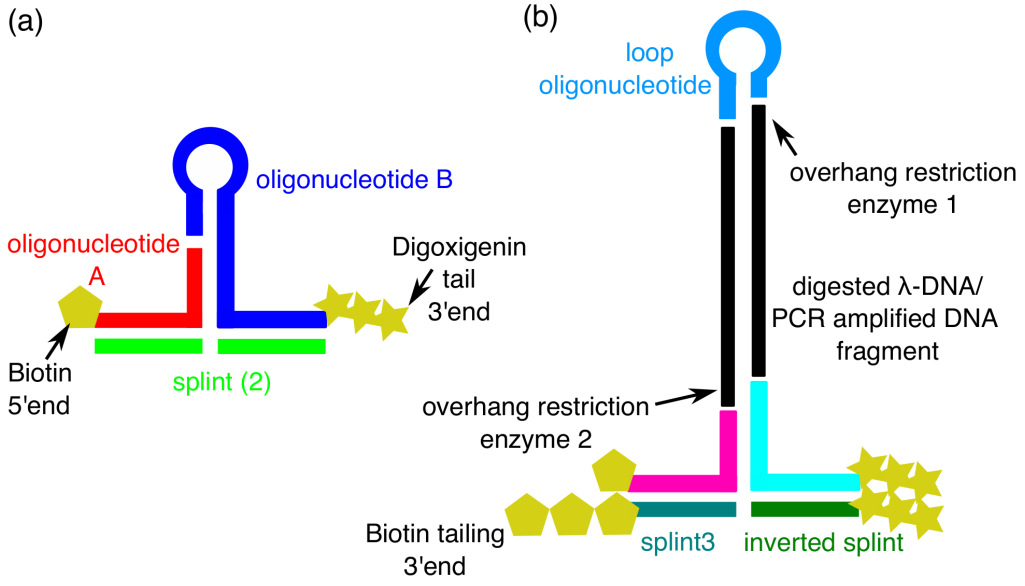

Figure 1: **Hairpin synthesis.** (a) The SH is assembled by ligating two oligonucleotides (blue and red). The oligonucleotide A is purchased biotinylated and the oligonucletide B is end-labelled with digoxigenins using the T4 terminal transferase. The splint oligonucletides are annealed to create dsDNA handles. (b) The LHs are assembled by ligating a set of oligonucleotides (magenta, cyan, blue) to the PCR-amplified and digested λ-phage fragment (black). Note that the complementary strands of the handles are also tailed. Color code as in Tables 1 and 2.

2 Oligonucleotides for hairpin synthesis

Name	Sequence
SH-A	5′-Biotin-AGT TAG TGG TGG AAA CAC AGT GCC AGC GCG AAC CCA CAA ACC GTG ATG GCT GTC CTT GGA GTC ATA CGC AA -3′
SH-B	5′-GAA GGA TG**G AAA AAA AAA AAA AAA AAA A**CA TCC TTC TTG CGT ATG ACT CCA AGG ACA GCC ATC ACG GTT TGT GGG TTC AGT TAG TGG TGG AAA CAC AGT GCC AGC GC-3′
splint	5′-GCG CTG GCA CTG TGT TTC CAC CAC TAA CT-3′

Table 1: Oligonucleotides used for the synthesis of the SH. The loop region is shown in bold.

Name	Sequence
13680b-loop	5′-Pho-GAT CGC CAG TTC GCG TTC GCC AGC ATC CGA **A** **CTA** CGG ATG CTG GCG AAC GCG AAC TGG C-3′
7138b-loop	5′-Pho-AAT TGC CAG TTC GCG TTC GCC AGC ATC CGA **A** **CTA** CGG ATG CTG GCG AAC GCG AAC TGG C-3′
4452b-loop	5′-Pho-TGA TAG CCT **ACT A**AG GCT ATC ACA TG-3′
1904b-loop	5′-Pho-CAT GAC AGT CGT TAG TAA CTA ACA TGA TAG TTA C**TT TT**G TAA CTA TCA TGT TAG TTA CTA ACG ACT GT-3′
964b-loop	5′-Pho-GTC ACT TAG TAA CTA ACA TGA TAG TTA C**TT** **TT**G TAA CTA TCA TGT TAG TTA CTA A-3′
700b-loop	5′-Pho-GTC ACT TAG TAA CTA ACA TGA TAG TTA C**TT** **TT**G TAA CTA TCA TGT TAG TTA CTA A-3′
Bio-cosRshort	5′-Bio-GAC TTC ACT AAT ACG ACT CAC TAT AGG GAA ATA GAG ACA CAT ATA TAA TAG ATC TT-3′
cosRlong	5′-Pho-GGG CGG CGA CCT AAG ATC TAT TAT ATA TGT GTC TCT ATT AGT TAG TGG TGG AAA CAC AGT GCC AGC GC-3′
Bio-cosLshort	5′-Bio-GAC TTC ACT AAT ACG ACT CAC TAT AGG GAA ATA GAG ACA CAT ATA TAA TAG ATC TT-3′
cosLlong	5′-Pho-AGG TCG CCG CCC AAG ATC TAT TAT ATA TGA GTC TCT ATT AGT TAG TGG TGG AAA CAC AGT GCC AGC GC 3′
HandBio-SMFP	5′-Bio-GAC TTC ACT AAT ACG ACT CAC TAT AGG GAA ATA GAG ACA CAT ATA TAA TAG ATC TTC GCA CTG AC -3′
HandDig-SMFP	5′-Pho-AAG ATC TAT TAT ATA TGT GTC TCT ATT AGT TAG TGG TGG AAA CAC AGT GCC AGC GC -3′
splint3	5′-TCC CTA TAG TGA GTC GTA TTA GTG AAG TC-3′
inverted-splint	3′-AAA AA-5′-5′-GCG CTG GCA CTG TGT TTC CAC CAC TAA C(SpC3)-3′

Table 2: Oligonucleotides used for the synthesis of long DNA hairpins. The loop region is shown in bold.

Name	Sequence
13680b − block − loop	5′-TAG TCG GAT GCT GGC GAA CGC GAA CTG GCG-3′
7138b − block − loop	5′-TAG TCG GAT GCT GGC GAA CGC GAA CTG GCG-3′
4452b − block − loop	5′-TAG TAG GCT ATC ACA TGC TGG CCA CCG GCT-3′
1904b − block − loop	5′-TTA CAA AAG TAA CTA TCA TGT TAG T-3′
964b − block − loop	5′-TTA CAA AAG TAA CTA TCA TGT TAG T-3′
700b − block − loop	5′-TTA CAA AAG TAA CTA TCA TGT TAG T-3′

Table 3: Blocking loop oligonucleotides used to generate ssDNA FECs for the different LHs.

References

[1] Alemany, A.; Ritort, F. Determination of the elastic properties of short ssDNA molecules by mechanically folding and unfolding DNA hairpins, Biopolymers **2014**, 101, 1193–1199.

[2] Camunas-Soler, J.; Manosas, M.; Frutos, S.; Tulla-Puche, J.; Albericio, F.; Ritort, F. Single-molecule kinetics and footprinting of DNA bis-intercalation: the paradigmatic case of Thiocoraline. Nucl. Acids Res. **2015**, 43, 2767–2779.

[3] Camunas-Soler, J.; Frutos, S.; Bizarro, C.V.; de Lorenzo, S; Fuentes-Perez, M.E.; Ramsch, R.; Vilchez, S.; Solans, C.; Moreno-Herrero, F.; Albericio, F., et al. Electrostatic binding and hydrophobic collapse of peptide–nucleic acid aggregates quantified using force spectroscopy, ACS Nano **2013**, 7, 5102-5113.
